# Risk and protective factors in academic burnout: exploring the mediating role of interpersonal emotion regulation in the link with social support

**DOI:** 10.3389/fpsyg.2025.1536951

**Published:** 2025-02-26

**Authors:** Irene Messina, Tatiana Rossi, Roberto Maniglio, Claudio Loconsole, Pietro Spataro

**Affiliations:** ^1^Deparment of Human and Social Sciences, Mercatorum University, Rome, Italy; ^2^Department of Engineering and Science, Mercatorum University, Rome, Italy

**Keywords:** interpersonal-emotion-regulation, social-support, venting, reassurance-seeking, academic-burnout, students-mental-health

## Abstract

Interpersonal Emotion Regulation (IER) may serve as a critical link between the established roles of social support and emotion regulation in mitigating academic burnout. This study explored the hypothesis that IER influences academic burnout through its impact on social support. 156 undergraduate students were involved in the study, with measures assessing academic burnout (Maslach Burnout Inventory—Student Survey), IER (Difficulties in Interpersonal Emotion Regulation), and social support (Multidimensional Scale of Perceived Social Support). Results confirmed the protective role of social support and revealed distinct effects of different IER forms. Specifically, reassurance-seeking emerged as a protective factor, positively predicting social support and indirectly reducing burnout levels. Conversely, venting was found to exacerbate burnout both directly and indirectly, by diminishing social support.

## Introduction

While initially studied in the context of workplace stress (Freudenberger, 1974), the concept of burnout has been adapted to educational environments. “Academic Burnout” (AB) refers to a condition marked by exhaustion from study demands, a cynical or detached attitude toward university tasks, and a sense of ineffectiveness in relation to academic performance (Schaufeli et al., 2002; Zhang et al., 2006). AB has become a widespread phenomenon among university students, with overall prevalence of each dimension of the syndrome estimated at 55.4% for emotional exhaustion, 31.6% for cynicism, and 30.9% for academic efficacy (Rosales-Ricardo et al., 2021). Students experiencing burnout are more likely to have low academic performance, and increased levels of stress and anxiety, which can lead to more severe issues such as substance abuse and suicidal ideation (Deeb et al., 2018; Kadhum et al., 2022; Dyrbye et al., 2008). Moreover, AB correlates with a heightened risk of extended academic timelines, delayed graduation, and even dropout (Rahmati, 2015; Madigan and Curran, 2021).

Social Support (SS) is commonly defined as the perception or experience of being cared for, valued, and part of a network of mutual assistance and obligations (Cobb, 1976). According to the stress-buffering hypothesis (Cohen and Wills, 1985), SS protects individuals from the harmful effects of stress by either mitigating the perception of stress or providing resources to effectively cope with it. This hypothesis has been extensively validated through numerous studies, which consistently demonstrate that SS serves as both a predictive and protective factor against burnout (House, 1981; Viswesvaran et al., 1999; Halbesleben, 2006; Kim et al., 2018). In the context of AB, a recent meta-analysis highlights that SS not only buffers stress but also enhances students’ ability to manage academic and practical challenges effectively (Kim and Lee, 2022), especially individuals’ subjective belief or perception of the availability of support from their social network (‘perceived social support’) (Bahar et al., 2024). Additionally, SS significantly influences the relationship between burnout and subjective well-being (Rehman et al., 2020).

The university is considered a high-stress context for students due to academic pressure (e.g., tight deadlines and high expectations), financial strain, social adjustments, and future uncertainty regarding career prospects (Beiter et al., 2015). In academic settings, as in other high-stress contexts, emotion regulation plays a crucial role in mitigating the impact of stress by shaping individuals’ emotional responses to challenging events (Chacón-Cuberos et al., 2019). Emotion regulation refers to the processes by which individuals influence the emotions they experience, when they experience them, and how they express and manage these emotions (Gross, 1998). A recent meta-analysis (Iuga and David, 2024) highlights emotion regulation as a significant factor in academic well-being, showing that adaptive strategies are negatively associated with AB, while maladaptive strategies can amplify the effects of stressful environments. Despite the importance of these previous findings, they neglect the role of interpersonal features of emotion regulation, that would be particularly relevant in association with SS. Interpersonal Emotion Regulation (IER) refers to efforts within social interactions in the pursuit of a regulatory goal, including all that ways by which individuals rely on others to regulate their emotion (Zaki and Williams, 2013). Previous contributions highlighted the relevance of IER as interconnection between SS and psychological suffering. For instance, Marroquín (2011) proposed a model built on substantial evidence highlighting the buffering role of SS in mitigating depression symptoms during adversity and suggested that IER strategies may mediate this protective effect. A recent study confirmed this hypothesis showing that the need to be soothed while regulating negative emotions may push people to seek SS, which can protect them from psychological distress (Gökdağ, 2021). Extending this hypothesis, we propose that IER may impact social support, which, in turn, could influence academic burnout. It is possible, however, that other forms of IER strategies may have different effects in the interplay between SS and AB. For instance, interpersonal venting and excessive reassurance-seeking are considered maladaptive IER strategies (Dixon-Gordon et al., 2018a; Messina et al., 2022), as they can lead to negative interpersonal outcomes, such as rejection, conflicts, or abandonment. These consequences may, in turn, reduce the availability of SS, exacerbating the risk of AB.

The aim of this study was to investigate the interplay between SS, IER, and AB among university students. Specifically, we first examined the statistical associations among these variables, with the expectation of confirming previous evidence that SS is negatively associated with AB. Second, we aimed to explore the relatively underexamined relationship between IER and AB, hypothesizing that greater use of IER strategies might correlate with higher levels of AB. Finally, we tested the hypothesis that IER mediates the relationship between SS and AB. Specifically, we propose that IER may shape perceptions or availability of social support, which, in turn, influences levels of academic burnout.

## Research methodology

### Participants and context

The study was conducted at Mercatorum University, a private Italian online university. The study sample included 156 undergraduate students (80 males, 76 females), with mean age 36.46(±11.14). The demographic characteristics of our participants are summarized in Table 1. Potential participants were invited electronically via email to students’ listservs. Questionnaires were administered using Google Forms, with access links distributed to students through the university psychological counseling service email list. This study received approval from the Ethical Committee for Psychological Research at Mercatorum University. Informed consent was obtained from all participants included in the study.

**Table 1:** Demographic characteristics of participants (*N* = 156).

Variable	Category	Frequency	Percentage
Gender	Female	76	48.72%
	Male	80	51.28%
Age group	21–30	63	40.38%
	31–40	38	24.36%
	41–50	35	22.44%
	51–60	18	11.54%
	61–70	1	0.64%
	> 70	1	0.64%
Family situation	Married or cohabiting with children	43	41.75%
	Married or cohabiting without children	28	27.18%
	In a relationship but living alone	32	31.07%
Work situation	Unemployed	28	17.95%
	Part-time employed	18	11.54%
	Full-time employed	110	70.51%
Education level	Business Management	37	23.72%
	Communication and Multimedia	28	17.95%
	Psychological Sciences and Techniques	21	13.46%
	Management Engineering	21	13.46%
	Legal Sciences	14	8.97%
	Gastronomy, Hospitality and Territories	12	7.69%
	Computer Engineering	7	4.49%
	Political Sciences and International Relations	7	4.49%
	Statistics and Big Data	5	3.21%
	Languages and Markets	3	1.92%
	Engineering of Infrastructures for Sustainable Mobility	1	0.64%

### Instruments

#### Maslach Burnout Inventory—Student Survey (MBI-SS)

The MBI-SS (Schaufeli et al., 1996) is a 15-item self-report questionnaire composed of three subscales: Exhaustion (e.g., “*I feel used up at the end of a day at university*”), Cynicism (e.g., “*I doubt the significance of my studies*”), and Professional Efficacy (e.g., “*During class I feel confident that I am effective in getting things done*”). We used the Italian version of MBI-SS from the study of Portoghese et al. (2018), in which reliability coefficients for each of the subscale scores were 0.86 for Exhaustion, 0.82 for Cynicism and 0.77 for Professional Efficacy.

#### Difficulties in interpersonal regulation of emotion (DIRE)

The DIRE is a scenario-based measure (Dixon-Gordon et al., 2018a), in which participants are invited to indicate, for each scenario, the likelihood that they would respond in the way described in each item, using a Likert scale ranging from 1 (“*very unlikely*”) to 5 (“*very likely*”). The DIRE allows the assessment of two forms of difficulties in interpersonal emotion regulation: Vent (e.g., “*Raise your voice or criticize your friends to express how you feel*”) and Reassurance-seek (e.g., “*Keep asking for reassurance*”). In the validation study of the Italian version of the DIRE good internal consistencies have been reported for both the Vent (*α* = 76) and Reassurance- Seek (*α* = 0.87) subscales (Messina et al., 2022).

#### Multidimensional scale of perceived social support (MSPSS)

The MSPSS (Zimet et al., 1988) is a self-report questionnaire composed by 12-item scored by using a 7-point Likert scale ranging from 1 (“*strongly disagree*”) to 7 (“strongly agree”). The items are organized in three subscales assessing different SS contexts: Family Support (e.g., “*My family really tries to help me*”), Friend Support (e.g., “*I can count on my friends when things go wrong*”), and Significant Other Support (e.g., “There is a special person with whom I share joys and sorrows”). Coefficient α for the Italian version in a previous study was 0.87 (Fabio and Kenny, 2012).

## Results

Table 2 reports descriptive statistics for the selected variables. It can be noted that the asymmetry and kurtosis values were always comprised between 1 and − 1, suggesting that the distributions of all variables were approximately normal (Tabachnick and Fidell, 1989). To formally test this assumption, we computed Mardia’s multivariate skewness and kurtosis coefficients, using the calculator provided by Cain et al. (2017). According to Bollen (1989), if Mardia’s coefficients are lower than *p*(*p* + 2), where *p* is the number of observed variables involved in the analysis, then the combined distribution of the variables is multivariate normal. In the present study, these coefficients were 4.02 for skewness and 46.87 for kurtosis, which were both safely lower than the threshold value (48).

**Table 2:** Descriptive statistics for the selected variables.

	Asimmetry	Kurtosis	M(SD)
Exhaustion (MBI-SS)	0.23 (0.19)	−0.50 (0.38)	2.21 (1.36)
Cynism (MBI-SS)	0.57 (0.19)	−0.49 (0.38)	1.63 (1–37)
Professional efficiency (MBI-SS)	0.07 (0.19)	−0.33 (0.38)	4.09 (0.66)
Reassurance-seeking (DIRE)	−0.01 (0.19)	−0.81 (0.38)	3.01 (1.12)
Venting (DIRE)	0.59 (0.19)	−0.19 (0.38)	2.09 (0.83)
Social support (MSPSS)	−0.78 (0.19)	0.21 (0.38)	15.60 (4.03)

Table 3 illustrates Pearson’s correlations. As can be noted: exhaustion and cynism were positively associated with Vent, but negatively associated with SS. Thus, as expected, students who used venting to a greater extent reported higher levels of exhaustion and cynism and lower levels of professional efficiency, whereas students who perceived greater SS reported lower levels of exhaustion and cynism and higher levels of professional efficiency. In addition, reassurance-seeking was positively associated with perceived social support, confirming that students who used this IER strategy to a greater extent were also more likely to perceive high social support. Lastly, reassurance-seeking and venting were positively correlated.

**Table 3:** Pearson’s correlations between all variables.

	1	2	3	4	5	6
1. Exhaustion	0.91					
2. Cynism	**0.54**	0.83				
3. Professional efficiency	**−0.21**	**−0.33**	0.80			
4. Reassurance-seeking	−0.04	−0.01	0.12	0.90		
5. Venting	**0.24**	**0.31**	**−0.16**	**0.16**	0.82	
6. Social support	**−0.30**	**−0.21**	**0.15**	**0.40**	−0.10	0.93

Cronbach’s α reliabilities are shown in the diagonal. Significant correlations are reported in bold (*p* < 0.05).

**Figure 1:**
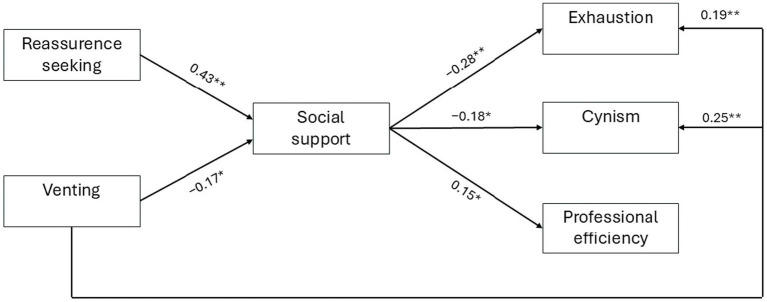
Final model of path analysis. Numbers refer to standardized paths (*p < 0.05; **p < 0.01).

To determine which variables predicted AB, we performed a path analysis using the *pathj* module of Jamovi, which is based on the lavaan R package (Rosseel, 2012) and also allowed us to test indirect mediation effects. Reassurance-seeking and venting were considered as the exogenous variable, SS as the endogenous mediator, and exhaustion, cynism and professional efficiency as the endogenous output variables. We began from a fully-saturated model which included all the predicted paths and iteratively stripped away non-significant paths, until we were left with a parsimonious model which showed an acceptable fit to the data – χ^2^(4) = 5.97, *p* = 0.20, CFI = 0.98, adj.GFI = 0.99, RMSEA = 0.056 [95% CI: 0.000–0.143, *p* = 0.37]. The model explained 19.1% of the variance in social support, 12.8% of the variance in exhaustion, 10.6% of the variance in cynism, and 2.4% of the variance in professional efficiency. As illustrated in Figure 1, the following paths were significant: (a) SS was positively predicted by reassurance-seeking (*β* = 0.43, *z* = 5.91, *p* < 0.001) and negatively predicted by venting (*β* = −0.17, *z* = −2.35, *p* = 0.019); (b) exhaustion and cynism were negatively predicted by SS (*β* = −0.28, *z* = −3.76, *p* < 0.001 and *β* = −0.18, *z* = −2.40, *p* = 0.017, respectively) and positively predicted by venting (*β* = 0.19, *z* = 2.59, *p* = 0.010 and *β* = 0.25, *z* = 3.46, *p* < 0.001, respectively); and (c) professional efficiency was positively predicted by SS (*β* = 0.15, *z* = 1.97, *p* = 0.049).

Most importantly, we also tested the significance of indirect effects via a bootstrapping procedure (5,000 iterations), using 95% confidence intervals. The consensus is that, if the confidence interval does not contain zero, then the indirect effect can be considered significant (Preacher and Hayes, 2004, 2008). The results (see Table 4) indicated that: (a) higher levels of reassurance-seeking predicted significant decreases in exhaustion and cynism by increasing SS (*β* = −0.12 [95%CI: −0.188, −0.041], *z* = −3.07, *p* = 0.002 and *β* = −0.07 [95%CI: −0.137, −0.002], *z* = −2.04, *p* = 0.040, respectively) and (b) higher levels of venting predicted significant increases in exhaustion by decreasing SS (*β* = 0.06 [95%CI: 0.000, 0.122], *z* = 1.97, *p* = 0.049). On the other hand, the positive indirect effect of reassurance-seeking on professional efficiency via increases in SS (*β* = 0.03 [95%CI: −0.031, 0.100], *z* = 1.01, *p* = 0.308) and the positive indirect effect of venting on cynism via decreases in SS (*β* = 0.04 [95%CI: −0.008, 0.082], *z* = 1.59, *p* = 0.110) did not reach the standard significance level, as well as the negative indirect effect of venting on professional efficiency via decreases in SS (*β* = −0.01 [95%CI: −0.056, 0.019], *z* = −0.94, *p* = 0.34).

**Table 4:** Direct, indirect and total effects estimated in the path analysis.

Predictor	Mediator	Output	β	z	p
Direct effects					
Reassurance-seeking	---	Exhaustion	0.04	0.53	0.591
Reassurance-seeking	---	Cynism	0.01	0.14	0.886
Reassurance-seeking	---	Professional efficiency	0.11	1.38	0.165
Venting	---	Exhaustion	0.24	2.64	0.008
Venting	---	Cynism	0.34	3.72	<0.001
Venting		Professional efficiency	−0.20	−2.13	0.032
Indirect effects					
Reassurance-seeking	Social support	Exhaustion	−0.12	−3.07	0.002
Reassurance-seeking	Social support	Cynism	−0.07	−2.04	0.040
Reassurance-seeking	Social support	Professional efficiency	0.03	1.01	0.308
Venting	Social support	Exhaustion	0.06	1.97	0.049
Venting	Social support	Cynism	0.04	1.59	0.110
Venting	Social support	Professional efficiency	−0.02	−0.94	0.343
Total effects					
Reassurance-seeking	---	Exhaustion	−0.08	−1.06	0.284
Reassurance-seeking	---	Cynism	−0.06	−0.85	0.390
Reassurance-seeking	---	Professional efficiency	0.14	2.01	0.043
Venting	---	Exhaustion	0.30	3.23	0.001
Venting	---	Cynism	0.38	4.13	<0.001
Venting		Professional efficiency	−0.22	−2.36	0.018

## Discussion

In line with prior research, our findings reaffirm the critical role of perceived SS in influencing students’ susceptibility to AB. SS emerged as a significant predictor across all dimensions of burnout, consistent with the well-established buffering hypothesis that emphasizes its protective role against stress-related outcomes (Cohen and Wills, 1985). Notably, the negative correlation between emotional exhaustion and total SS was stronger than the correlations observed for cynicism or professional efficacy. This suggests that emotional exhaustion - the most prominent and debilitating component of burnout—may be particularly sensitive to the availability and perception of social support, corroborating findings from earlier studies in work burnout (for a meta-analysis see Halbesleben, 2006) and AB (Rigg et al., 2013; Li et al., 2018).

Reassurance-seeking emerged as a protective factor, positively predicting SS and, through this increase, indirectly contributing to significant reductions in emotional exhaustion and cynicism while enhancing professional efficacy. As an interpersonal form of emotion regulation, reassurance-seeking involves actively seeking validation, comfort, or guidance from others during times of stress. This aligns with the hypothesis that IER strategies can strengthen social support, amplifying its protective effects against psychological distress (Marroquín, 2011; Gökdağ, 2021). Our findings are also in line with prior research demonstrating that reassurance-seeking fosters supportive relationships that provide both emotional sustenance and practical resources to navigate academic challenges (Kahn and Byosiere, 1992; Tripon, 2023). However, while reassurance-seeking appears beneficial in this context, caution is warranted given contrasting evidence highlighting potential risks if it becomes excessive or chronic. For example, the pursuit of constant validation from others, such as frequently asking for approval or reassurance about one’s appearance or performance, can undermine self-confidence and lead to emotional dependence (Dixon-Gordon et al., 2018b). Moreover, over time, excessive reassurance-seeking can strain relationships, lead to dependency (Joiner et al., 1999) and disrupt interpersonal networks for emotion regulation (Abe and Nakashima, 2022). These patterns can render reassurance-seeking interpersonally toxic and mark it as a potential behavioral indicator of risk for psychiatric concerns (Stewart and Harkness, 2015; Wakeling et al., 2020). Thus, while reassurance-seeking is valuable as an adaptive strategy, its long-term impacts and balance within relationships warrant careful consideration in future studies.

In contrast, venting appears to play a risk-enhancing role in students’ burnout. In our findings, venting had a dual negative impact on students’ burnout: one indirect, mediated by the loss of social support, and the other direct. Regarding the indirect influence, venting may erode the social networks that are crucial for buffering stress. Negative venting can lead to emotional contagion, where negative emotions are transferred to others, ultimately damaging relationships (Vijayalakshmi and Bhattacharyya, 2012; Niven et al., 2024). Indeed, venting may overwhelm the listeners, resulting in emotional fatigue and diminishing their capacity to offer supportive feedback. This weakening of SS further exacerbates burnout and emotional distress.

The direct effect of venting on emotional exhaustion and cynicism can be understood through the lens of cognitive neo-association theory (Berkowitz, 2012). According to this theory, venting may keep anger and frustration active in memory, reinforcing negative moods (Bushman, 2002). Previous research has linked the harmful effects of venting to a variety of mental health concerns, including depression (Malooly et al., 2017; Messina et al., 2023), borderline personality symptoms (Dixon-Gordon et al., 2018a, 2018b; Messina et al., 2022), and suicidal ideation (Chou et al., 2018). In the context of burnout, this mechanism may explain how venting exacerbates emotional exhaustion and cynicism in response to academic stressors, by perpetuating negative emotional states and hindering the recovery from stress.

This study has several limitations. First, due to the cross-sectional design of the current study, the temporal sequence of the independent variables, mediators, and dependent variables (causality) cannot be verified. This limitation is particularly relevant for reassurance-seeking, which emerged as a protective factor in this study, contrasting with previous evidence suggesting that excessive reassurance-seeking can have negative long-term consequences (Stewart and Harkness, 2015). To address this issue, future research could adopt a longitudinal design to better assess causal relationships and provide a more comprehensive understanding of how IER and SS influence AB among university students over time. Second, our sample was drawn from a single group of undergraduate students within one cultural context (Italy) and the specific setting of an online university, with students that may differ from those in traditional university environment (Pentina and Neeley, 2007). This unique composition may limit the generalizability of our findings to other student populations. Expanding research across diverse cultural and educational contexts is critical to enhancing the generalizability of findings. Future investigations into traditional versus online university settings or different cultural attitudes towards IER strategies can uncover nuanced insights. Finally, IER was assessed using a theory-driven instrument that focused exclusively on the dispositional use of reassurance-seeking and venting as emotion regulation strategies. This approach limits our ability to determine whether students employed other strategies to manage their emotions related to academic stress. Future studies using data-driven instruments or observational methods could provide a more comprehensive understanding of the range of strategies students use and identify which are most effective in mitigating AB.

## Conclusion

This study enhances our understanding of the interplay between social support, interpersonal emotion regulation, and academic burnout among university students. Our findings confirm the protective role of SS and underscore the significant mediating function of IER. Specifically, reassurance-seeking emerged as a positive interpersonal strategy, bolstering SS and reducing burnout, while venting acted as a maladaptive strategy that eroded SS and exacerbated burnout symptoms. These results align with existing literature on the stress-buffering effects of SS and suggest that fostering adaptive IER, particularly through reassurance-seeking, while minimizing the use of venting, could be an effective strategy to reduce burnout and improve academic well-being. This study provides valuable insights for clinical practice aimed at reducing AB and promoting mental well-being among university students. Based on our results, and in line with previous contributions (Tang et al., 2021; Messina et al., 2021; Messina et al., 2024), group psychological interventions targeting IER could be promising to contrast AB.

## Data Availability

The raw data supporting the conclusions of this article will be made available by the authors, without undue reservation.
